# Lats1/2 Differentially Modulate the Proliferative State of Dental Epithelial Progenitors and Ameloblasts in the Murine Incisor

**DOI:** 10.1111/ocr.70019

**Published:** 2025-08-29

**Authors:** Zhiyue Zhang, Shilpa Upadhyay, Colleen Luong, Wei Du, Jimmy K. Hu

**Affiliations:** ^1^ School of Dentistry University of California Los Angeles Los Angeles California USA; ^2^ State Key Laboratory of Oral Diseases and National Center for Stomatology and National Clinical Research Center for Oral Diseases, West China Hospital of Stomatology Sichuan University Chengdu Sichuan China; ^3^ Molecular Biology Institute University of California Los Angeles Los Angeles California USA

## Abstract

**Objective(s):**

Yes‐Associated Protein (YAP) is a critical regulator of cell proliferation and differentiation, having the capacity to convert differentiated cells into somatic stem cells in several contexts. Here we investigate the plasticity of adult mouse dental epithelial cells by testing the effects of ectopic YAP activation in dental epithelial progenitors and differentiated ameloblasts during incisor renewal.

**Materials and Methods:**

Using mice with dental epithelial deletion of *Lats1* and *Lats2*, which encode negative regulators of YAP, we assessed how ectopic YAP activation altered tissue structure, cell proliferation, and differentiation via histological analysis, EdU/BrdU labeling, and immunostaining. We also treated primary dental epithelial cells in 3D culture with TRULI, a LATS inhibitor, to test cell plasticity outside the context of an intact tissue.

**Results:**

YAP overactivation in the incisor epithelium induced ectopic cell proliferation in the apical bud and prolonged the proliferative state of dental epithelial progenitors, leading to a thickened suprabasal layer. However, ameloblasts remained non‐proliferative. Tissue structures and differentiation of pre‐ameloblasts were disrupted. Finally, LATS inhibition was able to induce colony formation in some dissociated, 3D‐cultured ameloblasts.

**Conclusion:**

YAP activation promotes the expansion of dental epithelial progenitors but not ameloblasts in vivo, suggesting irreversible differentiation during tooth renewal. However, when dissociated, some ameloblasts acquire plasticity. This offers a strategy to manipulate proliferation in dental epithelial cells under different conditions and may have broader applications in orthodontics, where a modular control of progenitors and differentiated cells is critical for ensuring safe stem cell‐based treatments.

## 1 Introduction

Tissue homeostasis and injury repair rely on the activity of resident somatic stem cells, which have the capability to both self‐renew and give rise to differentiated cell types. This balance between self‐renewal and differentiation is tightly regulated to maintain stem cell populations while ensuring a continuous supply of specialised cells essential for tissue function. Disruptions in this balance can lead to hyperplasia if self‐renewal is excessive [1] or impaired tissue maintenance if differentiation is aberrant [2]. Notably, certain somatic tissues exhibit cellular plasticity, enabling differentiated cells to revert to a progenitor‐like state, such as during injury repair [3]. A central goal in stem cell biology is thus to elucidate the mechanisms governing these processes and their roles in tissue regeneration.

**FIGURE 1:**
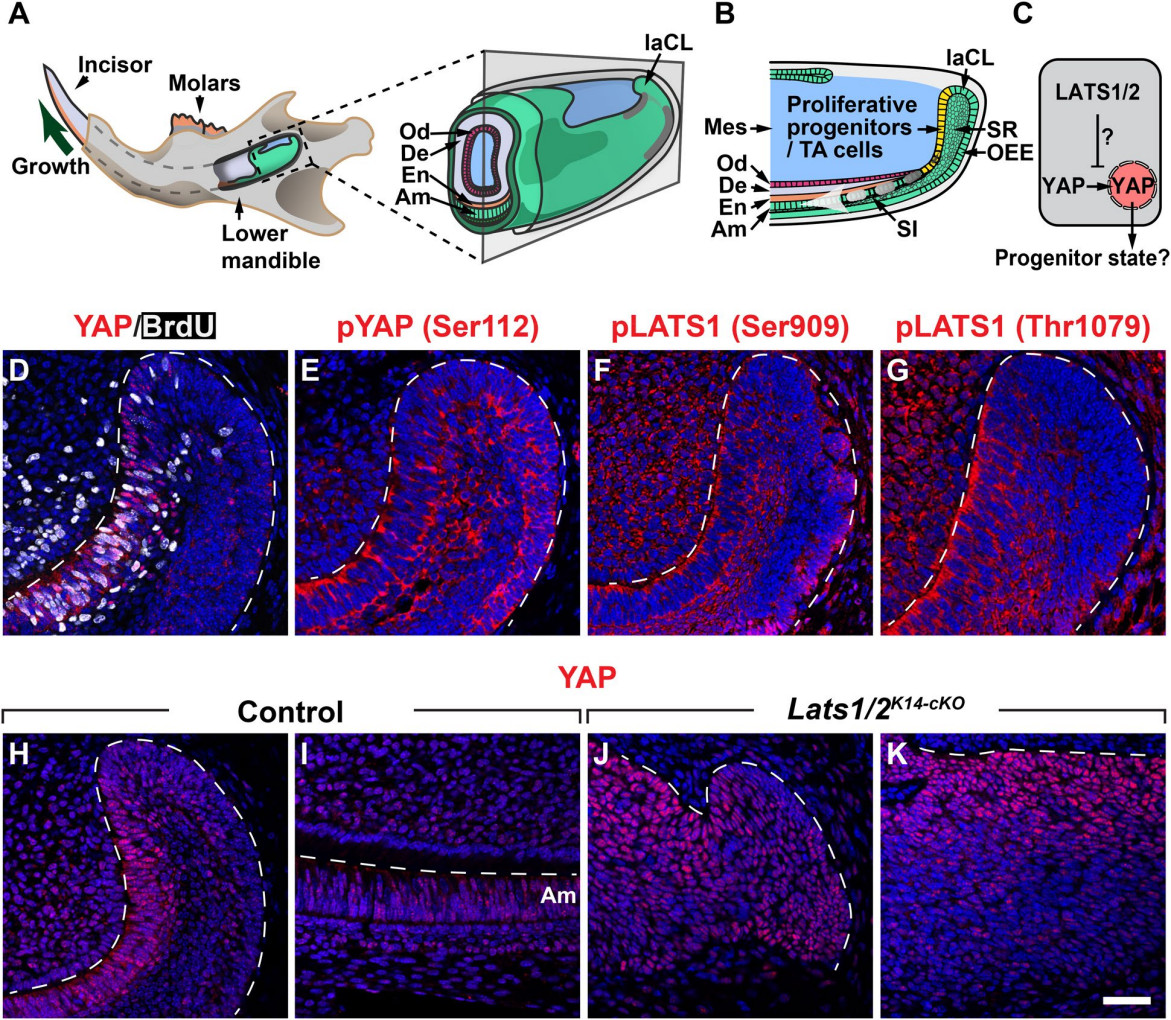
LATS1/2 suppress nuclear localization of YAP in the dental epithelium. (A, B) Schematics of the mouse incisor. Dental epithelial stem cells and proliferative transit‐amplifying (TA) progenitors reside in the labial cervical loop (laCL) and give rise to enamel (En)‐secreting ameloblasts (Am) in the basal layer and the stratum intermedium (SI) and stellate reticulum (SR) cells in the suprabasal layer. De, dentin; Od, odontoblast; OEE, outer enamel epithelium. (C) Here we investigate the role of LATS1/2 in controlling YAP localization within the incisor epithelium and whether YAP activation alone can drive epithelial cells into a progenitor‐like state. (D–G) Expression of YAP, pYAP (Ser112), pLATS1 (Ser909), and pLATS1 (Thr1079) in the laCL. BrdU labels the proliferative population in (D). (H–K) Expression of YAP in the laCL (H, J) or the ameloblast region (I, K) of control and *Lats1/2* ^*K14‐cKO*^ mutant epithelia on day 10 after CreER induction. Dashed lines outline the incisor epithelium. Representative images are shown. Scale bar in (K) represents 40 μm in (D–K).

The continuously growing adult mouse incisor provides a useful, tractable model for studying stem cell biology [4, 5]. Unlike human teeth, mouse incisors grow continuously due to the maintenance of dental epithelial stem cells (DESCs) in the labial cervical loop, a stratified epithelial niche structure at the apical end of the tooth (Figure 1A) [6]. Within the basal layer, DESCs first give rise to a group of transitory and highly proliferative progenitors, called transit‐amplifying (TA) cells, which in turn produce a steady supply of enamel‐secreting ameloblasts (Figure 1B) [7, 8]. This process resembles a conveyor belt‐like movement and enables continuous replacement of aged, apoptosed distal cells and the renewal of enamel lost through incisal wear. Concurrently, TA cells also generate stratum intermedium (SI) and stellate reticulum (SR) cells in the suprabasal layer [9]. This hierarchical and spatially defined arrangement of cells thus allows researchers to explore the incompletely understood mechanisms that regulate the maintenance, proliferation, and lineage commitment of different cell populations within a self‐renewing epithelial tissue. Uncovering the regulatory pathways that govern lineage specification and cell plasticity in the mouse incisor will also provide key insights for deriving and maintaining human dental epithelial cells, which are largely lost after tooth eruption [10].

A key regulator of cell proliferation and differentiation is Yes‐associated protein (YAP), a transcriptional co‐factor negatively regulated by the Hippo signalling pathway [11]. When the Hippo pathway is activated, signals relayed through the MAP4K/MST1/2‐LATS1/2 kinase cascade phosphorylate YAP at several serine residues, including Ser112 and Ser381 in mice (or Ser127 and Ser397, respectively, in humans) [12, 13]. This results in the sequestration of YAP in the cytoplasm and its degradation, effectively inactivating YAP. Conversely, when Hippo signalling is inactive, YAP remains unphosphorylated and accumulates in the nucleus, allowing it to drive the transcription of downstream genes critical for promoting cell proliferation and/or modulating differentiation [14]. Therefore, deletion of *Yap* disrupts proliferation–dependent tissue homeostasis and impairs injury repair, while deregulation of Hippo signalling often leads to YAP overactivation and tissue hyperplasia [15, 16, 17]. Notably, induced YAP activation can reprogramme differentiated somatic cells isolated from mouse tissues, including the mammary gland, brain, and pancreas, into proliferative tissue‐specific progenitor cells [18, 19]. Similarly, deletion of *Lats1/2* in the mouse mammary luminal epithelium enables luminal cells to acquire basal stem cell characteristics, [20] demonstrating that YAP can confer progenitor fate plasticity to differentiated cells in certain contexts.

In the mouse incisor, we have previously shown that YAP and its homologue TAZ are required for maintaining proper cell proliferation in the dental epithelial TA population [21]. In contrast, deletion of *Lats1/2* led to dental epithelial overgrowth. However, the temporal progression of the hyperplastic phenotype has not been thoroughly studied. It is also unclear whether different cell populations within the dental epithelium exhibit differential susceptibility to YAP overactivation. Here, we address these questions and investigate the extent of cellular plasticity in differentiated ameloblasts as influenced by YAP activity (Figure 1C). Our findings show that *Lats1/2* deletion and YAP overactivation enhances proliferation of progenitor cells and production of suprabasal cells but does not drive ameloblast proliferation in the intact tissue. However, LATS1/2 inhibition promotes colony formation of isolated ameloblasts in 3D culture, indicating that YAP‐driven progenitor fate plasticity in ameloblasts is context‐dependent.

## 2 Materials and Methods

### 2.1 Ethics Statement

All animal procedures were conducted in compliance with animal protocols approved by the Anonymized Committee.

### 2.2 Mouse Lines and Induction of Alleles

*K14* ^*CreER*^ [22], *K14* ^*Cre*^ (MGI: 2680713) [23], *Sox2* ^*CreER*^ (MGI: 5298207) [24], *Lats1* ^f/f^ (MGI: 5568586), *Lats2* ^f/f^ (MGI: 5568589) [25], *R26* ^*mT/mG*^ (MGI: 3716464) [26], *R26* ^*rtTA*^ (MGI: 3584521) [27], and *YAP* ^*S127A*^ [15], were used to generate *K14* ^*CreER*^; *Lats1* ^*f/f*^; *Lats2* ^*f/f*^, *Sox2* ^*CreER*^; *Lats1* ^*f/f*^; *Lats2* ^*f/f*^, *K14* ^*Cre*^; *R26* ^*rtTA*^; *YAP* ^*S127A*^, and *K14* ^*Cre*^; *R26* ^*mT/mG*^ mouse lines. Mice were group housed and genotyped as previously published. Both male and female mice at 8 weeks of age were used. For CreER activation, tamoxifen dissolved in corn oil (5 mg/30 g body weight/day) was delivered by oral gavage for 2 consecutive days. For rtTA activation, 1 mg/mL of doxycycline dissolved in 5 mg/mL of sucrose water was provided to animals. For 5‐ethynyl‐20‐deoxyuridine (EdU) and Bromodeoxyuridine (BrdU) incorporation, 100 μL of EdU or BrdU (10 mg/mL) was delivered through intraperitoneal injection at various timepoints as indicated in results and figures. Mice were euthanised by CO_2_ followed by cervical dislocation for sample collection at different timepoints as specified in results and figures.

### 2.3 Tissue Preparation and Staining

Euthanized mice were perfused with PBS and 4% paraformaldehyde (PFA) in PBS. Dissected lower mandibles were then fixed in 4% PFA overnight at 4°C. For paraffin sections, mandibles were dehydrated through serial ethanol washes and equilibrated to paraffin using Leica ASP300S. Paraffin embedded mandibles were sectioned at 7 μm. Haematoxylin & Eosin (H&E) staining and immunostaining of paraffin sections were carried out as previously described [28]. Primary antibodies against amelogenin (ab153915, Abcam), BrdU (B35128, Thermo), cleaved Notch1 intracellular domain (NICD) (4147, Cell Signalling), Keratin 17/19 (12434, Cell Signalling), Ki67 (ab15580, Abcam), P‐cadherin (13‐2000Z, Thermo), pLATS1 (Ser909) (9157, Cell Signalling), pLATS1 (Thr1079) (bs‐7913R, Bioss), pYAP (Ser127) (4911, Cell Signalling), and YAP (4912, Cell Signalling) were used (1:100 dilution). Keratin 17/19 and Ki67 were detected using secondary antibodies conjugated with Alexa fluorophores (Thermo). The rest of the antibodies were detected using biotinylated secondary antibodies and amplified using ABC HRP Kit (Vector Laboratories) and Tyramide (PerkinElmer). H&E images were taken using a Leica DM1000 microscope. Fluorescent images were acquired using either an Olympus IX81 microscope or a Leica SP8 confocal microscope. Image analysis was carried out using tools and plugins in Fiji. Fluorescence signal areas and epithelial lengths were quantified using the Measure function. The average epithelial thickness was measured using the InteredgeDistance v3 macro. EdU/BrdU‐labelled cells were counted using the Cell Counter plugin.

### 2.4 3D Culture of Dental Epithelial Cells

Cervical loops and ameloblast regions of the incisor epithelium were separately isolated from *K14* ^*Cre*^ *; R26* ^*mT/mG*^ mice and dissociated into single cells as previously described [29]. Cells were then mixed with growth factor‐reduced Matrigel (356231 Corning) and split into two groups, one cultured with control DMSO and the other with 10 μM LATS inhibitor TRULI [30]. The culture media contain DMEM/F12 (GIBCO), 20 ng/mL EGF (R&D), 25 ng/mL bFGF (R&D), 1X B27 (GIBCO), and 1% pen/strep. Cells were cultured for 5 days and spheroids were counted and imaged. 2 mice were used for each experiment and 3 experiments were conducted.

### 2.5 Statistical Analysis

All experiments were replicated at least three times, and no data were excluded. Representative images were shown in figures. Quantifications were displayed as mean ± SD (standard deviation) in graphs. Unpaired two‐tailed Student's *t*‐test or one‐way ANOVA followed by Tukey's HSD test was used to calculate *p* values. Significance was set at *p* < 0.05 with a confidence interval of 95%. *p < 0.05; **p < 0.01; ***p < 0.001; ****p < 0.0001.

## 3 Results

### 3.1 Active Hippo Signalling Suppresses YAP Overactivation in the Incisor Epithelium

To determine the activity of YAP and Hippo signalling in the adult mouse incisor epithelium, we examined the expression of YAP, phosphorylated YAP (pYAP), and phosphorylated LATS1 (pLATS1) by means of immunostaining. This showed that progenitor cells within the proliferative TA region contain high levels of nuclear YAP (Figure 1D), confirming our previous finding that active YAP maintains TA proliferation [21]. In contrast, cells in the outer enamel epithelium and in the suprabasal stellate reticulum layer display minimal nuclear YAP localization, as proliferation is typically low in these regions. Using an antibody targeting the inactive, cytoplasm‐restricted phospho‐YAP (pYAP (Ser112)), we found that pYAP is present throughout the cervical loop, including the proliferative zone (Figure 1E), indicating that YAP activity is actively modulated in the incisor epithelium to maintain homeostasis. Consistent with this result, the activated, phosphorylated LATS1 (pLATS1 (Ser909 and Thr1079)) is expressed in the epithelium, with the highest expression in the proliferative zone (Figure 1F,G). Hippo signalling is thus active in the incisor epithelium, permitting inhibition of YAP.

We next tested the functional requirement of LATS1/2 in regulating YAP activity and incisor epithelium homeostasis by utilising the *K14* ^*CreER*^; *Lats1/2* ^*f/f*^ (*Lats1/2* ^*K14‐cKO* (*conditional knockout*)^) mouse model, in which the inducible CreER recombinase is expressed specifically in the incisor epithelium, enabling targeted deletion of both *Lats1* and *Lats2* upon tamoxifen administration (Figure S1A–C). We also generated *Lats1* ^*K14‐cKO*^ and *Lats2* ^*K14‐cKO*^ single mutants. While single deletion yielded no discernible phenotypes (Figure S1D,E), deletion of both *Lats1* and *Lats2* resulted in hyperplasia and expansion of the dental epithelium in *Lats1/2* ^*K14‐cKO*^ mice 10 days after the initial CreER activation (*n* = 6/6), when compared to CreER‐negative littermate controls (Figure 1H–K). Ectopic nuclear YAP expression could also be observed in the outer enamel epithelium, stellate reticulum, and many cells within the presumptive ameloblast region (Figure 1H–K). These findings demonstrated that LATS1/2 are required for suppressing nuclear YAP localization and preventing uncontrolled tissue growth in the dental epithelium, consistent with their established roles in other tissues.

### 3.2 Deletion of *Lats1/2* Drives Tissue Hyperplasia and Expansion of Suprabasal Cells

**FIGURE 2:**
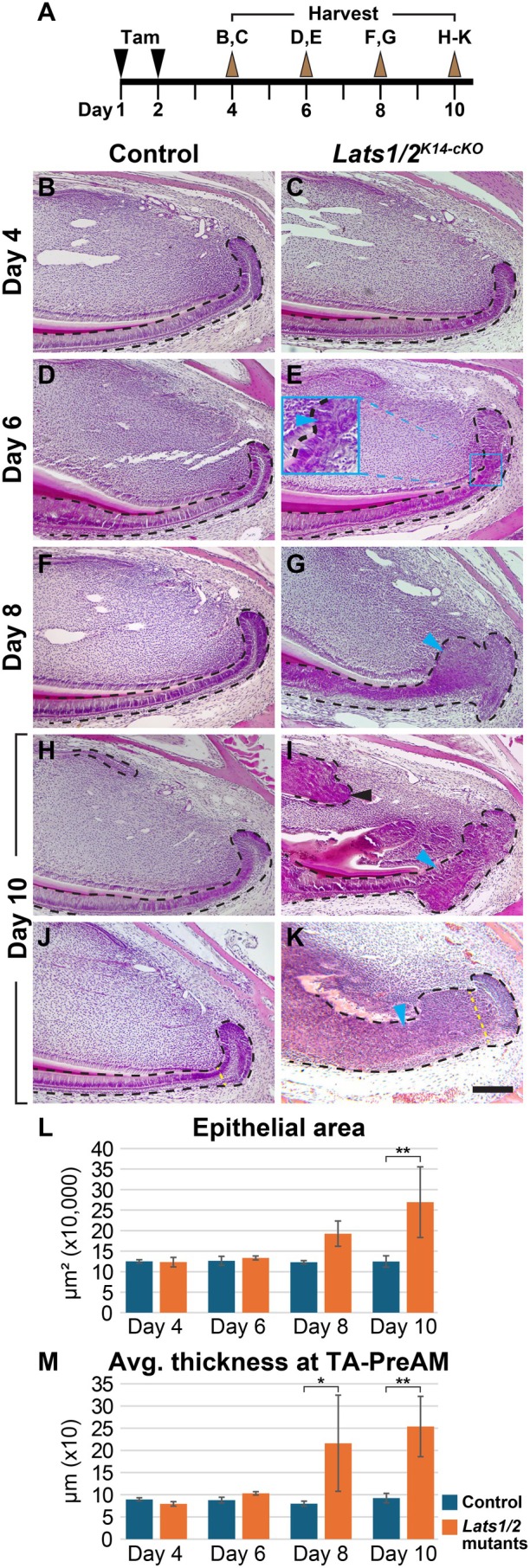
Deletion of *Lats1/2* results in a gradual expansion of the dental epithelium. (A) Timeline depicting CreER induction by tamoxifen (Tam, black arrowheads) and sample collection (brown arrowheads). (B–K) H&E staining of control and *Lats1/2* ^*K14‐cKO*^ mutant dental epithelia at different timepoints following CreER activation. Blue arrowheads indicate the expanded epithelial regions in mutants. The zoomed‐in area in (E) is shown in the blue box. Black arrowhead in (I) indicates the expanded lingual cervical loop. (L) Quantification of the epithelial area within the outlined labial epithelium (*n* = 3). (M) Quantification of the average epithelial thickness at the transit‐amplifying (TA)‐pre‐ameloblast (PreAM) region, as indicated by the yellow dashed line (*n* = 3). Dashed black lines outline the incisor epithelium. Representative images are shown. All quantitative data are presented as mean ± SD. The *p* values were determined using one‐way ANOVA followed by Tukey's HSD test (*p < 0.05, **p < 0.01). Scale bar in (K) represents 200 μm.

The *Lats1/2* ^*K14‐cKO*^ mice provided a valuable model for investigating cell plasticity in the dental epithelium, specifically for exploring the ability of YAP to drive differentiated cells towards a proliferative progenitor‐like state. To begin, we sought to establish the temporal progression of the hyperplastic phenotype and performed H&E staining of control and *Lats1/2* ^*K14‐cKO*^ mutant incisors on day 4, 6, 8, and 10 after the initial CreER induction (Figure 2A). While we did not observe histological changes before day 4 (Figure 2B,C), small epithelial swellings appeared in the TA region on day 6 (Figure 2D,E). By day 8, large expansions extended from the TA and pre‐ameloblast regions into the mesenchyme, however the distal ameloblast region maintained its normal morphology (Figure 2F,G). On day 10, the epithelium showed significant thickening, and tissue overgrowth was clearly visible in all or parts of the ameloblast region (Figure 2H–M). Similarly, deletion of *Lats1/2* using *Sox2* ^*CreER*^, which is expressed in dental epithelial progenitors in the apical cervical loop [31], also resulted in epithelial expansion (Figure S2). Together, these findings indicate that the *Lats1/2* deletion‐induced tissue overgrowth begins in the proliferative progenitor cell region of the epithelium and gradually extends towards the distal ameloblast region.

**FIGURE 3:**
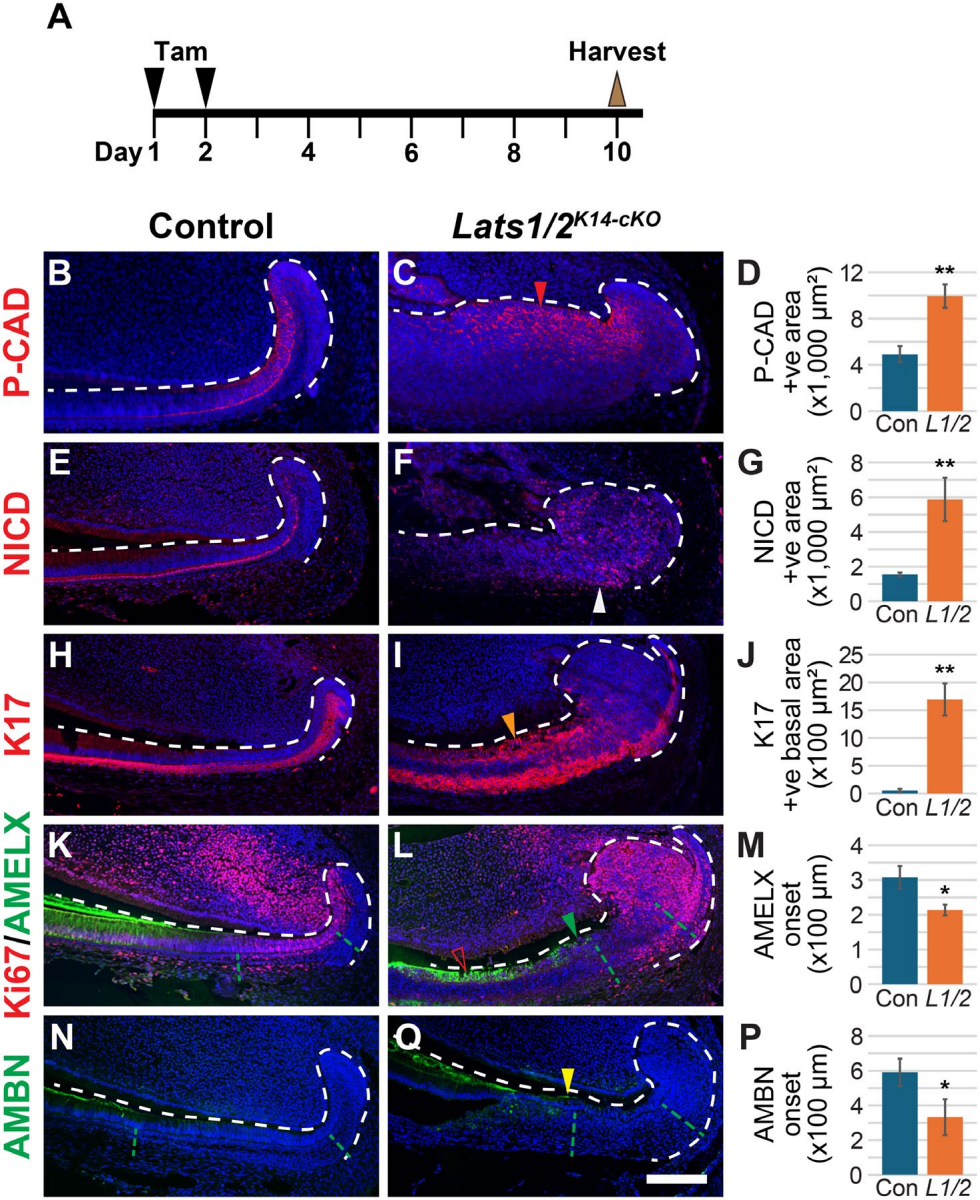
Cell organisations and identities are disrupted in the *Lats1/2* ^*K14‐cKO*^ mutant epithelium. (A) Timeline depicting CreER induction by tamoxifen (Tam, black arrowheads) and sample collection (brown arrowheads). (B–D) Expression of P‐CAD, a marker for TA cells, is expanded along with the epithelial overgrowth in *Lats1/2* ^*K14‐cKO*^ mutants (red arrowhead). Quantification shows P‐CAD‐positive areas in the dental epithelium. (E–G) Expression of NICD, labeling SI cells, is also expanded in the *Lats1/2* ^*K14‐cKO*^ mutant epithelium (white arrowhead). Quantification shows NICD‐positive areas in the dental epithelium. (H–J) Expression of K17, a suprabasal cell marker, becomes ectopically expressed in the mutant basal cells (orange arrowhead). Quantification shows K17‐positive areas in the epithelial basal layer. (K–P) Images showing the expression of amelogenin (AMELX) and ameloblastin (AMBN), which label ameloblasts, and Ki67, which labels mitotic cells in control and *Lats1/2* ^*K14‐cKO*^ mutant dental epithelia. Green arrowhead in (L) indicates ectopic AMELX expression in the same K17‐overexpressing region in (I). Images in (I, L) are from serial sections. Open red arrowhead in (L) denotes the absence of proliferating cells in ameloblasts. Yellow arrowhead in (O) marks apically extended AMBN expression. Quantifications show the distance measured from the epithelial bend to the onset of AMELX or AMBN expression (i.e., between green dashed lines). White dashed lines outline the incisor epithelium. Representative images are shown. All quantitative data are presented as mean ± SD. The *p* values were determined using Student's *t*‐test (*p < 0.05, **p < 0.01). Scale bar in (O) represents 130 μm in (B, C) and 200 μm in (E–O).

To determine which cell populations were expanded in the *Lats1/2* ^*K14‐cKO*^ mutant epithelium, we analysed the expression of several known markers [9]. This revealed that TA cells, marked by P‐cadherin (P‐CAD), SI cells, expressing cleaved Notch1 intracellular domain (NICD), and suprabasal cells, labelled by Keratin 17 (K17) all constituted the thickened epithelium (Figure 3A–J). Furthermore, while these markers were confined to specific regions within the control epithelium, their organisation was disrupted in the *Lats1/2* ^*K14‐cKO*^ mutant incisors. For example, NICD was no longer restricted to a single line of SI cells (Figure 3F), and K17 was ectopically expressed in numerous basal cells outside the cervical loop (Figure 3I). We also observed that the expression of amelogenin, which typically begins in secretory‐stage ameloblasts, had shifted towards the apical bud and extended into the region with ectopic K17 expression (Figure 3K–M). Similarly, the onset of ameloblastin expression was initiated earlier along the *Lats1/2* ^*K14‐cKO*^ mutant epithelium (Figure 3N–P). These findings thus suggest that the kinetics of ameloblast differentiation were altered, and cell identities became less distinct in the *Lats1/2* ^*K14‐cKO*^ mutant epithelium. Importantly, the cell proliferation marker Ki67 was only observed in the expanded cervical loop region and was absent in amelogenin‐expressing cells (Figure 3K,L), indicating that differentiated ameloblasts remained non‐proliferative in the *Lats1/2* ^*K14‐cKO*^ mutants.

### 3.3 *Lats1/2* Deletion Promotes Proliferation in Progenitor Cells but Not Differentiated Cells

**FIGURE 4:**
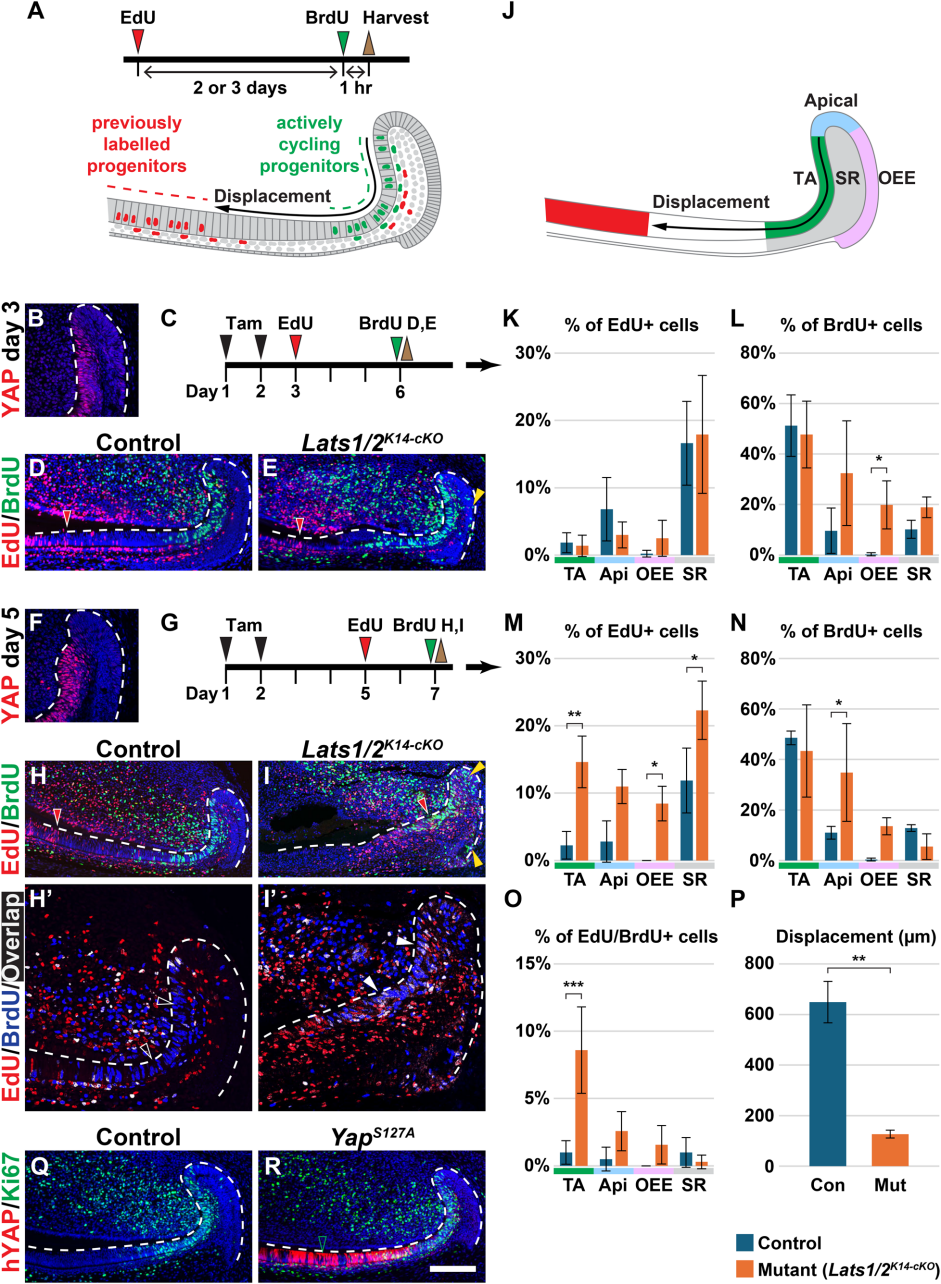
Loss of *Lats1/2* in the incisor epithelium extends the proliferative state of progenitors but does not induce proliferation in ameloblasts. (A) Schematic illustrating EdU/BrdU dual labeling in the incisor dental epithelium. EdU incorporation allows tracking of labelled progenitors for 2 or 3 days and assessment of their displacements until tissue collection. BrdU incorporation reports the proliferation pattern at the time of tissue collection. (B) Representative YAP expression on day 3. (C) Timeline depicting CreER induction by tamoxifen (Tam), EdU injection on day 3, and BrdU injection with sample collection on day 6. (D, E) Distribution of EdU‐ and BrdU‐labelled cells in control and *Lats1/2* ^*K14‐cKO*^ dental epithelia using the scheme shown in (C). (F) Representative YAP expression on day 5. (G) Timeline depicting CreER induction by tamoxifen (Tam), EdU injection on day 5, and BrdU injection with sample collection on day 7. (H, I) Distribution of EdU‐ and BrdU‐labelled cells in control and *Lats1/2* ^*K14‐cKO*^ dental epithelia using the scheme shown in (G). Red arrowheads mark the proximal end of the population labelled by EdU in the basal layer in (D, E, H, I), and yellow arrowheads mark the ectopic cycling cells in mutants in (E, I). (H′, I′) Enlarged views of panels (H) and (I). Open and closed white arrowheads indicate the absence or presence of EdU/BrdU double‐labelled cells in the control or *Lats1/2* ^*K14‐cKO*^ TA regions respectively. Grey signals are overlapping EdU/BrdU labels. (J) Schematic depicting the four zones where the percentage of EdU‐labelled, BrdU‐labelled, and EdU/BrdU double‐labelled cells were quantified, as well as the measurement of distance for EdU cell displacement. (K–N) Quantification of the % of EdU‐labelled (EdU+) or BrdU‐labelled (BrdU+) cells in different regions of the dental epithelium, when EdU and BrdU labeling is performed on day 3 and 6 (*n* = 5) (K, L) or on day 5 and 7 (*n* = 3) (M, N). (O) Quantification of the % of EdU/BrdU double‐labelled (EdU/BrdU+) cells in different regions of the dental epithelium when EdU and BrdU labeling is performed on day 5 and 7 (*n* = 3). (P) Quantification of EdU cell displacement between day 5 and 7 (*n* = 3). (Q, R) Expression of Ki67 and the constitutively active human YAPS127A in control and YAPS127A samples. Open green arrowhead indicates the absence of proliferation in cells with overactivated YAP. Dashed lines outline the incisor epithelium. Representative images are shown. All quantitative data are presented as mean ± SD. The *p* values were determined using one‐way ANOVA followed by Tukey's HSD test (K‐M) or Student's *t*‐test (N) (*p < 0.05, **p < 0.01, ***p < 0.001). Scale bar in (P) represents 100 μm in (B, F), 130 μm in (D, E, H, I, O, P), and 65 μm in (H′, I′).

The results above prompted us to further examine how *Lats1/2* deletion in *Lats1/2* ^*K14‐cKO*^ affects the kinetics of cell proliferation and displacement. To that end, we performed EdU/BrdU double labeling, in which EdU was injected 48 or 72 h before sacrifice to label and lineage‐trace proliferative progenitors, and BrdU was administered 1 h before sacrifice to assess the proliferation patterns of actively cycling progenitor cells (Figure 4A,J). We first compared cell kinetics between controls and *Lats1/2* ^*K14‐cKO*^ mutants at a stage when phenotypic changes had not yet emerged, and YAP expression was relatively unaffected, likely due to LATS1/2 proteins persisting after their genetic deletion (Figure 4B). To do this, we injected EdU on day 3 and BrdU on day 6 (Figure 4C). This showed comparable displacement of progenitor cells that are labelled by EdU on day 3 in both the control and *Lats1/2* ^*K14‐cKO*^ mutant basal layers, as the majority of EdU‐labelled basal cells had moved to the distal epithelium and only a few EdU‐positive basal cells remained in the cervical loop (Figure 4D,E,K). EdU‐labelled cells were also observed in the distal SI region and in the deeper suprabasal layer, as displaced by their proliferative, BrdU‐positive counterparts in the cervical loop (Figure 4D,E,L). Notably, when these samples were collected on day 6, some cells in the mutant outer enamel epithelium had begun to proliferate (Figure 4E,L). However, most of the EdU‐labelled cells were BrdU‐negative (Figure 4E), suggesting that these EdU‐labelled cells had likely differentiated and ceased proliferating. This observation is also consistent with our result above showing that *Lats1/2* deletion was unable to drive proliferation in differentiated cells.

Next, we targeted progenitor cells that had begun to overexpress YAP and undergone expansion by injecting EdU on day 5 and lineage‐tracing them for 2 days until BrdU injection and tissue harvesting on day 7 (Figure 4F,G). Surprisingly, the displacement of EdU‐labelled mutant basal cells in *Lats1/2* ^*K14‐cKO*^ was significantly less than that of the controls (Figure 4H,I,P), and many cells were retained in the TA region with BrdU co‐labeling, suggesting that they remained proliferative (Figure 4H′,I′,M–O). There were also more EdU‐labelled cells in the mutant suprabasal layer (Figure 4I,M), which were likely derived from the proliferating basal cells, given that the number of actively dividing SR cells (BrdU‐labelled) was similar between controls and *Lats1/2* ^*K14‐cKO*^ mutants (Figure 4N). Concurrently, proliferation expanded into the apical cervical loop and the outer the epithelium, while distal epithelium stayed non‐proliferative (Figure 4I,N). Collectively, these results demonstrate that *Lats1/2* deletion prolongs the progenitor state but is insufficient to revert differentiated ameloblasts to progenitors. Consistent with this result, direct overexpression of a constitutively active YAP (YAPS127A) in the ameloblast region also failed to induce proliferation in these cells (Figure 4Q,R).

### 3.4 LATS Inhibition Induced Proliferation in Some Primary Differentiated Dental Epithelial Cells

**FIGURE 5:**
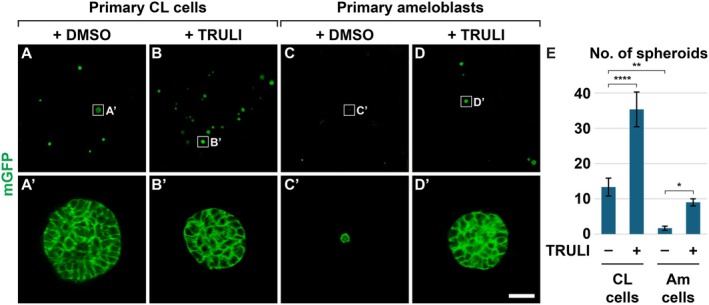
Inhibition of LATS1/2 function promotes spheroid formation in some primary differentiated cells. (A–D) Dissociated primary cells from either the cervical loop (CL) or ameloblast regions were cultured in 3D with or without the LATS inhibitor, TRULI. Proliferative cells form spheroid colonies. (A′–D′) Enlarged views of selected spheroids or single cells in (A–D). (E) Quantification of total spheroid numbers per experiment in different conditions (*n* = 3). Representative images are shown. All quantitative data are presented as mean ± SD. The *p* values were determined using one‐way ANOVA and Tukey's HSD test (*p < 0.05, **p < 0.01, ****p < 0.0001). Scale bar in (D′) represents 400 μm in (A–D) and 30 μm in (A'–D′).

One potential mechanism preventing YAP‐driven proliferation in differentiated dental cells is the presence of local inhibitory signals, such as contact inhibition or inputs from other signalling pathways. To test this, we dissociated wild type cells from either the cervical loop or the ameloblast region and then evenly divided the isolated single cells for 3D culture with either DMSO vehicle control or the LATS inhibitor, TRULI [30]. Consistent with our findings from genetic deletion of *Lats1/2*, pharmacological inhibition of LATS significantly increased the number of spheroids formed from dissociated cervical loop progenitor cells (Figure 5A,B,E). Notably, while differentiated cells formed very few spheroids under control conditions, LATS inhibition enhanced their spheroid‐forming capacity (Figure 5C–E), suggesting that some differentiated cells can be induced to proliferate when cultured as primary cells.

## 4 Discussion

The maintenance of tissue homeostasis relies on the precise control of resident progenitor cell proliferation. Here, we show that LATS1/2 are key regulators of YAP activity in the adult mouse dental epithelium, crucial for limiting the duration of the proliferative state within the progenitor pool and preventing uncontrolled epithelial growth. Our mouse model of epithelial *Lats1/2* deletion also allowed us to probe the progenitor fate plasticity in differentiated ameloblasts. We found that dental progenitors and ameloblasts responded differently to LATS1/2 loss, and YAP overactivation alone was insufficient to induce ameloblast proliferation in vivo. However, upon dissociation, LATS inhibition was able to induce proliferation in some 3D‐cultured primary ameloblasts. Therefore, YAP's ability to drive proliferation in adult incisors is both cell type and context dependent.

In our study, the deletion of *Lats1/2* led to increased nuclear YAP and a progressive expansion of the dental epithelium, originating from the proliferative TA region. This anti‐proliferative function of LATS is thus consistent with their established roles in other tissues [32]. Importantly, sequential labeling of proliferative progenitors using EdU and BrdU at two different time points revealed that in the *Lats1/2* ^*K14‐cKO*^ mutants, the displacement of TA cells was drastically reduced, with many TA cells remaining in the TA region and continuing to proliferate. In contrast, TA cells in control animals completely exited the TA region and became postmitotic after 48 h. Loss of LATS1/2 thus prolongs the proliferative state of dental epithelial progenitors. At first glance, it may seem counterintuitive that prolonged proliferation would result in reduced cell displacement in the basal layer. However, we also observed a significant increase in EdU‐labelled cells in the suprabasal SR region, which remained low‐proliferating and maintained the same low % of BrdU+ cells as the control. Therefore, the most logical explanation is that *Lats1/2* deletion promoted stratification, driving more basal cells into the suprabasal layer. This would lead to fewer cells being retained in the basal layer and consequently decreased cell displacement. Interestingly, LATS1/2 have been shown in other contexts to suppress epithelial‐mesenchymal transition (EMT) and cell migration by inhibiting NFκB and TGFβ signalling [32, 33, 34]. As epithelial stratification and/or overgrowth involve aspects of EMT, it will be important in the future to determine whether loss of LATS1/2 drives epithelial expansion through a similar mechanism.

Our study also revealed significant disruptions in cellular organisation and marker expression in the *Lats1/2*‐deficient dental epithelium. For example, the SI marker NICD was no longer restricted to a single line of cells adjacent to the basal layer but rather, throughout the expanded epithelium. Additionally, the suprabasal marker K17 became ectopically expressed in the basal layer that also expressed amelogenin. Therefore, while *Lats1/2* ^*K14‐cKO*^ mutant progenitors are capable of differentiation, the identities of their progeny are blurred, and a clear distinction between different basal and suprabasal cell populations is lost. In most other stem cell systems, inactivation of LATS1/2 or overactivation of YAP sustains the proliferative state of progenitor cells and inhibits their differentiation [35, 36, 37]. However, in a few cases, loss of LATS1/2 instead triggers precocious differentiation or aberrant fate specification [38, 39]. Our findings suggest that LATS1/2 also play a role in ensuring the correct specification of basal cells in the mouse incisor, and an important question for future research is to elucidate how LATS1/2 regulate the specification of different lineages and their organisation within the dental epithelium. Approaches such as single cell RNA‐sequencing and spatial transcriptomics will help elucidate this important question.

Besides promoting progenitor cell proliferation, YAP has also been shown to have the capacity to revert differentiated cells back to a progenitor state in cell culture, as well as to impart basal stem cell characteristics to suprabasal cells in vivo [18, 19, 20]. However, we found that neither *Lats1/2* deletion nor direct expression of a constitutively active YAPS127A was able to induce proliferation in differentiated ameloblasts. These findings thus indicate that active YAP alone was insufficient to confer progenitor fate plasticity to differentiated dental epithelial cells. While the exact mechanism preventing ameloblast dedifferentiation remains unclear, ameloblasts are typically tightly packed and receive signals from the neighbouring mesenchyme and stratum intermedium, including BMP4, FGF, and EGF [40]. It is therefore possible that contact inhibition along with the local signalling microenvironment plays critical roles in maintaining the ameloblasts in a differentiated state, creating a barrier that cannot be overridden solely by YAP activation. Consistent with this notion, we observed that when cells isolated from the ameloblast region were dissociated and cultured as single cells in 3D Matrigel, treatment with the LATS inhibitor TRULI significantly enhanced their proliferation and induced colony formation to a level that is comparable to those produced by progenitor cells from the cervical loop region. We thus conclude that at least some of the differentiated dental epithelial cells exhibit plasticity when removed from the context of an intact tissue.

## 5 Conclusion

This study highlights the complexity of progenitor maintenance and differentiation within the dental epithelium, where LATS1/2 serve not only as gatekeepers of progenitor proliferation but also as key regulators of cell fate specification. Our findings also reveal ameloblasts' differential resistance to YAP‐driven proliferation in tissues and in culture, uncovering the pivotal role of the tissue microenvironment in modulating YAP and Hippo signalling functions. This underscores the importance of considering these factors when developing therapeutic strategies to target YAP for the regeneration of dental or other craniofacial tissues.

## Author Contributions

Conceptualization: J.K.H. Methodology: Z.Z., W.D., and J.K.H. Formal analysis: Z.Z., S.U., C.L., and J.K.H. Investigation: Z.Z., S.U., and C.L. Data curation: Z.Z., S.U., and C.L. Writing – original draft: S.U. and J.K.H. Writing – review and editing: Z.Z., S.U., C.L., W.D., and J.K.H. Supervision: J.K.H. Project administration: J.K.H. Funding acquisition: J.K.H.

## Ethics Statement

All animal procedures were conducted in compliance with animal protocols approved by the UCLA Institutional Animal Care and Use Committee (Protocol Number ARC‐2019‐013).

## Conflicts of Interest

The authors declare no conflicts of interest.

## Supporting information


**Appendix S1:** ocr70019‐sup‐0001‐AppendixS1.pdf.

## Data Availability

The data that support the findings of this study are available from the corresponding author upon reasonable request.
