# Supplementary material for: Lats1/2 Differentially Modulate the Proliferative State of Dental Epithelial Progenitors and Ameloblasts in the Murine Incisor
Source: Orthod Craniofac Res. 2025 Aug 29;28(Suppl 1):S59–69. doi: 10.1111/ocr.70019 (PMC12690751; doi:10.1111/ocr.70019)
Supplement: Supplementary file 1 — Appendix S1: ocr70019‐sup‐0001‐AppendixS1.pdf. [file OCR-28-S59-s001.pdf]

**SUPPLEMENTAL FIGURES**

**Supplemental Figure S1**

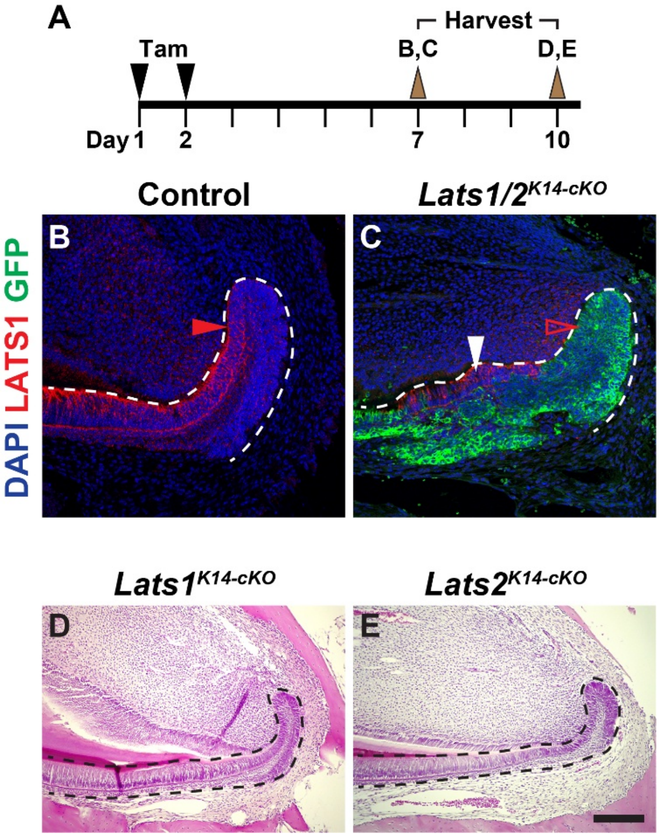


**Supplemental Figure S1. Deletion of both *Lats1* and *Lats2* are required to induce epithelial hyperplasia.** (**A**) Timeline depicting CreER induction by tamoxifen (Tam, black arrowheads) and sample collection (brown arrowheads). (**B**,**C**) *Lats1/2*$^{K14\text{-}cKO}$ mutant epithelium becomes expanded. Immunostaining shows LATS1 expression in the control incisor epithelium (red arrowhead) and its successful deletion in *Lats1/2*$^{K14\text{-}cKO}$ mutants (open red arrowhead), specifically in cells with CreER-mediated recombination. Membrane GFP is a Cre reporter. Due to the mosaic nature of tamoxifen-induced CreER activity, a few uninduced cells remain GFP-negative and retain LATS1 expression (white arrowhead). (**D**,**E**) Single deletion of *Lats1* (*Lats1*$^{K14\text{-}cKO}$) or *Lats2* (*Lats2*$^{K14\text{-}cKO}$) does not result in epithelial hyperplasia (n = 5). Dashed lines outline the incisor epithelium. Scale bar in (E) represents 100 μm in (B,C) and 200 μm in (D,E).

**Supplemental Figure S2**

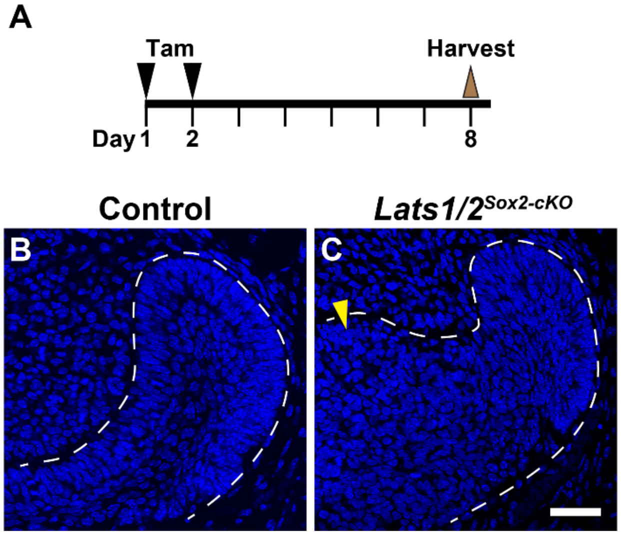


**Supplemental Figure S2. Deletion of *Lats1/2* in *Sox2*-expressing progenitors resulted in epithelial hyperplasia.** (**A**) Timeline depicting CreER induction by tamoxifen (Tam, black arrowheads) and sample collection (brown arrowheads). (**B**,**C**) DAPI staining of control (B) and *Lats1/2*$^{Sox2\text{-}cKO}$ (C) laCLs. Yellow arrowhead indicates the expanded mutant epithelium. Dashed lines outline the incisor epithelium. Scale bar represents 40 µm.
